# Innovative HBV Animal Models Based on the Entry Receptor NTCP

**DOI:** 10.3390/v12080828

**Published:** 2020-07-30

**Authors:** Jochen M. Wettengel, Benjamin J. Burwitz

**Affiliations:** 1 Institute of Virology, Technische Universität München/Helmholtz Zentrum München, Trogerstr. 30, 81675 Munich, Germany; wettengel@tum.de; 2 Vaccine & Gene Therapy Institute, Oregon Health & Science University, 505 N.W. 185th Avenue Beaverton, Tanasbourne, OR 97006, USA

**Keywords:** sodium taurocholate co-transporting polypeptide, hepatitis B virus, rhesus macaque, animal model

## Abstract

Hepatitis B is a major global health problem, with an estimated 257 million chronically infected patients and almost 1 million deaths per year. The causative agent is hepatitis B virus (HBV), a small, enveloped, partially double-stranded DNA virus. HBV has a strict species specificity, naturally infecting only humans and chimpanzees. Sodium taurocholate co-transporting polypeptide (NTCP), a bile acid transporter expressed on hepatocytes, has been shown to be one of the key factors in HBV infection, playing a crucial role in the HBV entry process in vitro and in vivo. Variations in the amino acid sequence of NTCP can inhibit HBV infection and, therefore, contributes, in part, to the species barrier. This discovery has revolutionized the search for novel animal models of HBV. Indeed, it was recently shown that variations in the amino acid sequence of NTCP represent the sole species barrier for HBV infection in macaques. Here, we review what is known about HBV entry through the NTCP receptor and highlight how this knowledge has been harnessed to build new animal models for the study of HBV pathogenesis and curative therapies.

## 1. Introduction

Hepatitis B virus (HBV) is the causative agent of hepatitis B, an acute and in some cases chronic infectious liver disease. The World Health Organization (WHO) estimates that worldwide more than two billion people have been infected with the virus and 257 million people are living with a chronic HBV infection [1]. These chronically-infected patients have increased risks of developing liver cirrhosis, liver failure or hepatocellular carcinoma [2]. With approximately 887,000 deaths yearly, the disease and its consequences are a major global health problem, prompting the WHO to announce a program to eliminate hepatitis B by 2030 [3]. However, despite more than 50 years of research, there are no reliable curative treatments against chronic HBV infection, so new innovative therapies are urgently needed. Unfortunately, many molecular mechanisms of the viral replication cycle, especially those of viral entry, are not fully understood, hindering construction of crucial in vivo models. Here, we discuss what is known about early events in HBV infection and how this knowledge has paved the way for the generation of innovative animal models for the study of HBV pathogenesis and curative therapies.

## 2. Hepatitis B Virus (HBV)—Molecular Biology and Mechanisms of Hepatocyte Entry

HBV is a relatively small, enveloped DNA virus with a diameter of 42 nm and the prototype of the Hepadnaviridae family. The infectious virion, also called a Dane particle, consists of an icosahedral nucleocapsid bearing a 3200 nucleotide-long viral DNA genome that is bound to the HBV polymerase. The nucleocapsid is enveloped by a lipid bilayer membrane in which three types of transmembrane surface proteins are embedded [4]. These HBV surface proteins are classified as large (L), middle (M), or small (S) proteins. While the M protein consists of the S protein with an N-terminal extension (termed PreS2), the L protein consists of the M protein with an N-terminal extension (termed PreS1) [5]. Notably, these surface proteins are shared with the hepatitis D virus (HDV), a virusoid or so-called “satellite virus”, that uses HBV surface proteins for its own envelope [6]. Due to these shared envelope proteins, HDV is often used as a surrogate model for studying HBV entry [7]. Although both viruses presumably have the same or similar processes in uptake, HDV capsids and genomic RNA follow alternate replication pathways after cellular entry [8].

The HBV replication cycle can be categorized into early and late steps. While late steps like genome replication, virion assembly and particle release are quite well understood and summarized in various reviews [9,10,11], many aspects of the early steps involving HBV liver targeting, cellular entry and viral uncoating are still cryptic [12]. Factors involved in these early steps are listed in Table 1 and discussed below.

HBV is a highly infectious hepatotropic virus, and its replication is strictly restricted to hepatocytes. In fact, an inoculum of a few virions is sufficient to cause a full HBV infection in chimpanzees, suggesting highly specific liver targeting and infectivity of the virus [32]. However, it remains largely unknown how HBV targets hepatocytes in vivo. Recently, Esser et al. showed that HBV liver accumulation was diminished in ApoE knock-out mice, indicating specific HBV liver transport along the lipoprotein remnant pathway (Table 1) [13]. Indeed, there is strong evidence for a direct HBV-ApoE association, since ApoE-specific antibodies are capable of blocking HBV infection and capturing HBV particles similar to anti-HBs antibodies [33]. Although both studies support ApoE as a mediator directing HBV to the liver, other factors and receptors may also play a role in its hepatotropism.

After reaching the liver, HBV virions attach to heparan sulfate proteoglycans, in particular glypican 5, on the basolateral side of hepatocytes (Table 1) [14]. However this low-affinity interaction does not explain HBV hepatotropism since heparan sulfate proteoglycans are expressed on many other cell types in vivo. Indeed, the identity of a high-affinity HBV entry receptor on the surface of hepatocytes remained elusive for many decades.

Over the past 10 years, significant strides have been made in identifying the critical factors, receptors, and inhibitors of HBV binding and entry (Table 1). It was long known that myristoylated synthetic peptides comprising the N-terminal stretch of PreS1 inhibit HBV infection of primary hepatocytes, presumably by binding a specific HBV receptor [34,35]. Myrcludex B, a synthetic myristoylated peptide of amino acids 2–48 of PreS1, has been particularly valuable, since it can be used for evaluation of HBV binding on hepatocytes originating from different species. Indeed, early studies showed that Myrcludex B specifically binds human and tupaia primary hepatocytes in vitro, but also to HBV non-susceptible hepatocytes from mice, rats and dogs. However, it does not bind to pig, cynomolgus macaque, or rhesus macaque hepatocytes, suggesting an absent or altered receptor in these species [36]. In accordance with this, Myrcludex B accumulates in vivo in the livers of mice, rats and dogs, but not in cynomolgus macaques, suggesting this particular receptor is both hepatocyte and species-specific [37]. Using this knowledge of peptide binding, Yan et al. utilized a tagged, myristoylated PreS1 peptide on tupaia hepatocytes to identify the sodium taurocholate co-transporting polypeptide (NTCP), which they went on to show is a key factor in HBV binding and entry (Table 1) [17].

NTCP is broadly expressed across many species. However, there are significant differences in the amino acid sequence between species (66.2% amino acid conservation across sequenced mammals) [38]. Human NTCP is a 349 amino acid-long transmembrane glycoprotein that is encoded by the *SLC10A1* gene [39]. Its expression is predominantly restricted to hepatocytes where the protein physiologically acts as a bile acid transporter, responsible for the uptake of conjugated bile acids from the bloodstream through an anion exchange mechanism [40,41]. NTCP has a very short half-life of less than 24 h and its expression is rapidly downregulated in isolated primary hepatocytes in vitro [42,43]. This down-regulation is responsible for reduced or blocked HBV infection in long-term cultured primary human hepatocytes and most immortalized human hepatoma cell lines [44]. Therefore, overexpressing human NTCP on these cells is necessary to study HBV infection in vitro [45]. Although the presence or absence of NTCP distinguishes the ability of HBV to infect hepatocytes, the exact mechanism of its function in the early HBV replication cycle, aside from binding HBV, is not fully understood [46]. A recent study suggests that following heparin sulfate proteoglycan attachment, HBV binds to NTCP which subsequently oligomerizes prior to viral entry [26]. This is in accordance with Chakraborty et al., who showed that while HBV cell attachment is NTCP independent (via heparin sulfate proteoglycans), expression of NTCP has a significant effect on HBV internalization, indicating that the initial HBV-NTCP interaction takes place prior to cellular entry [31]. This cellular entry has been identified as clathrin-depended, suggesting a clathrin-mediated endocytosis of HBV and NTCP [30]. Indeed, Iwamoto et al. identified epidermal growth factor receptor (EGFR) as a host-entry cofactor to NTCP, forming an HBV-NTCP-EGFR complex mediating HBV internalization [28]. Using siRNA-mediated EGFR knockdown, the authors could specifically inhibit HBV internalization, but saw no effect on HBV attachment or genome replication. Interestingly, HBV internalization could also be inhibited through an NTCP decoy peptide comprising amino acids 131–150, suggesting this region as crucial for the formation of the HBV-EGFR-NTCP complex. In addition, Iwamoto et al. showed in a follow-up study that activation and auto-phosphorylation of EGFR by EGF triggers a time-dependent re-localization of the PreS1 peptide to the early and late endosomes as well as to lysosomes, suggesting a transport of incoming HBV along the endosomal network [47]. This finding is in accordance with Macovei et al. who showed that Rab5 and Rab7, both factors involved in the endocytic pathway in early and late endosomes, are imperative for a successful infection [48].

Interestingly, HBV genotype D has a deletion of 11 amino acid in the N-terminal PreS1 domain compared to other HBV genotypes. Recently, Murayama et al. have shown that this deletion provides an advantage in HBV attachment and infection when introduced into HBV genotype C [49], indicating differences in hepatocyte binding and internalization between HBV genotypes. A similar result was confirmed in a recent study showing that N-terminal deletions of 15 or 18 amino acids in the PreS1 domain enhanced infectivity in genotype B and C [50].

After internalization, the processes of uncoating and nuclear transport are largely unknown, however it is assumed that HBV uncoating takes place in the early or late endosome and that the capsid is transported along the microtubule network to the nucleus where the capsids or the rcDNA are released through the nuclear pore into the nucleus [51,52].

As mentioned above, HBV-NTCP interaction can be antagonized by small peptides like Myrcludex B [18] but also by small molecules (Table 1) and thereby blocking HBV infection. However, a functional HBV-NTCP interaction can also be prevented by sequence polymorphisms in NTCP itself, indicating that species-specificity of HBV is, in part, determined by NTCP sequence.

There are two amino acid regions in human NTCP known to play a crucial role in HBV binding and post-binding entry, both of which are required for a productive infection (Table 2). Variations to amino acids 84–87 (RLKN) can drastically decrease or even block infection [53]. Interestingly, variations in this region do not affect HBV-NTCP binding, indicating that this region is important for an as yet unknown post-binding receptor function. In contrast, amino acids 157–165 (KGIVISLVL) have been identified as necessary for effective HBV-NTCP binding [17]. In fact, a single NTCP polymorphism at amino acid G158R blocks HBV binding and subsequent infection, however it does not block NTCP’s physiological function as a bile acid transporter [17,54]. Indeed, this polymorphism has been reported in many Old-World monkeys, and recent studies imply that this amino acid substitution appeared due to positive selection, potentially due to pressure by a viral infection [38,55].

Taken together, these data indicate that polymorphisms in the NCTP sequences might restrict HBV species-tropism in other species, leading to the question of whether additional animal models of HBV infection, especially Old World monkeys, could be constructed by exogenous expression of human NTCP or humanization of the endogenous NTCP.

## 3. The Role of the Sodium Taurocholate Co-Transporting Polypeptide (NTCP) in HBV Animal Models

Although many mechanisms of the HBV replication cycle can be studied using readily available in vitro cell culture models, complex questions like HBV pathogenesis, carcinogenesis or immunogenesis need to be addressed within the context of an in vivo animal model. Over the last few decades, several different animal models have been utilized to study HBV. Early work focused on ducks, woodchucks, and woolly monkeys, which are natural hosts to HBV-related viruses within the Hepadnaviridae family [56,57,58]. While these animal models allowed researchers to answer basic questions about viral replication and pathogenesis, the genetic and structural variability between HBV and these related viruses precluded meaningful studies focused on immunogenesis and drug development. Therefore, researchers set out to identify new animal models permissive to experimental HBV infection.

Humans and chimpanzees are the only natural hosts for HBV, and this strict species-tropism is now understood to be due, in part, to sequence variability within the NTCP of other species. However, NTCP sequences that deviate from human NTCP but still support HBV infection have been discovered. For instance, the northern treeshrew (*Tupaia belangeri*) is permissive to experimental HBV infection despite several amino acid differences in both functional regions 84–87 and 157–165 of NTCP (Table 2) [59]. Unfortunately, the differences in the immune system, a paucity of species-specific reagents, as well as the low rate of infectivity limit a broad use of treeshrews in HBV research [60].

Mice (*Mus musculus*) are an often used and well-studied animal model with a fast reproduction rate and well characterized immunity. Notably, amino acids 157–165 of mouse NTCP, are capable of binding HBV, but variations across amino acids 84–87 block HBV infection completely (Table 2) [53]. Although humanization of region 84–87 or expression of human NTCP in transgenic mice renders them permissive to HDV infection, HBV infection is restricted, indicating that other, yet unknown, post-entry factors inhibit a full HBV infection cycle in mice hepatocytes [61,62]. Therefore, mice with chimeric humanized livers, repopulated with human hepatocytes, are currently used to study HBV infection in vivo, however, these mice are immunodeficient and important immunological questions cannot be addressed [63,64]. In response to this, multiple groups are now pursuing chimeric humanized liver mice with transplanted human immune systems [65,66].

Recently, Lempp et al. showed that NTCP is the limiting host factor for HBV infection of pig (*Sus Scrofa*), cynomolgus macaque (*Macaca fascicularis*), and rhesus macaque (*Macaca mulatta*) hepatocytes in vitro [67]. In contrast, hepatocytes from mice, rats, and dogs expressing human NTCP could not support a full HBV replication cycle in this study, suggesting post-binding blocks to entry or replication. Therefore, pigs and macaques are currently the most promising candidates for new immunocompetent HBV animal models.

While the functional regions for HBV binding and post-binding entry in pig NTCP have not yet been characterized, it is known that macaque NTCP possesses the same two functional regions as human NTCP and encodes the aforementioned G158R polymorphism within amino acids 157–165 (Table 2), which abrogates HBV binding [17,54]. Therefore, we extended the Lempp et al. study to show that expression of a chimeric human-macaque NTCP (humanized at amino acids 157–165 or 158 alone) renders rhesus macaque hepatocytes permissive to a full HBV replication cycle in vitro (unpublished data). In addition, we showed that vector-mediated expression of human NTCP in rhesus macaque livers renders them permissive to HBV infection in vivo [68]. This discovery has significant implications, particularly given the moratorium on chimpanzee research enacted in 2011 by the National Institutes of Health and other international health agencies [69]. Indeed, these findings make rhesus macaques a highly promising species for translational HBV research [68].

Interestingly, New World monkeys including marmosets, woolly-, squirrel-, spider- or capuchin-monkeys do not possess the G158R mutation in NTCP and may be capable of supporting HBV infection [71]. Indeed, in woolly monkeys (Lagothrix lagotricha), another hepadnavirus species, the woolly monkey HBV (WMHBV), was found, suggesting that other New World monkeys may also support hepadnavirus replication [58,70]. In support of this, Chen et al. experimentally infected squirrel monkeys (Saimiri boliviensis boliviensis) with WMHBV, however, squirrel monkeys were not permissive to HBV, indicating other host limiting factors for different hepadnaviruses [71]. Recently, Souza et at. found a new hepadnavirus in capuchin monkeys (Sapajus xanthosternos) that also uses NTCP as entry receptor, indicating that capuchin monkeys may also be permissive to WMHBV and perhaps even HBV [72]. Taken together, New World monkeys are also a promising avenue for building new HBV models, although many of these species are endangered an there remains no evidence that New World monkeys can be infected experimentally with HBV.

## 4. Rhesus Macaques as a New HBV Animal Model

There are multiple advantages using rhesus macaques in HBV research. First, rhesus macaque physiology, particularly that of the liver, is extremely similar to humans. Second, the rhesus macaque immune system is highly similar to humans and reagents to phenotype and modulate the immune response are expansive, particularly compared to all other HBV models outside of the mouse [73]. Third, the HIV/SIV model in rhesus macaques has led to studies elucidating the optimal intramuscular dosing of the same reverse transcriptase inhibitors that have been shown to be effective in HBV infection [74]. Fourth, the ability to longitudinally sample the blood, liver, spleen, and lymph nodes gives much more resolution on the effects of HBV treatment and ongoing pathology and immunogenesis. Indeed, we have collected blood weekly and liver biopsies monthly for over a year in rhesus macaques. Finally, rhesus macaques are purpose-bred for research at multiple international primate centers and are, therefore, readily available. Overall, the rhesus macaque model holds significant promise as a translational HBV animal model.

We pioneered the first rhesus macaque model, showing in 2017 that exogenous expression of human NTCP on hepatocytes facilitates HBV infection in vivo [68]. This model was characterized by low levels of HBV in the serum persisting for up to 7 weeks, T cell responses against all HBV proteins, and expression of HBV core antigen in a small percentage of hepatocytes. Limitations of this first approach were: (1) inadequate transduction of rhesus macaque hepatocytes with Ad- and AAV-based viral vectors expressing human NTCP, (2) transient viremia in multiple infected animals, lasting as short as 2 weeks, and (3) lack of detectable HBe and HBs antigens in the serum, most likely due to the low-level HBV replication observed.

We have significantly improved the rhesus macaque model since 2017 by addressing the shortfalls observed in our initial publication. Specifically, we have: (1) improved transduction of rhesus macaque hepatocytes with Ad-based vectors by optimizing the dose and introducing pharmaceutical suppression of reactivity to high-dose viral vector administration, (2) increased the level of HBV replication (>10^7^ copies/mL serum) and duration of HBV infection (>1 year post-challenge), and (3) shown that all measurements of human HBV infection are present in our model, including ALT flares, HBe and HBs antigens in serum, and localized HBV RNA in rhesus macaque hepatocytes by immunofluorescence. Overall, we have built a robust non-human primate model of HBV infection through exogenous expression of human NTCP. We are now testing currently approved clinical treatments for HBV in our model, after which time we will begin testing pre-clinical drugs.

Finally, the use of viral vectors and the potential immunogenicity of exogenous human NTCP expression represent confounding variables that may have unintended effects in the model. Therefore, we are currently using CRISPR/Cas9 editing of embryos to generate transgenic rhesus macaques that express humanized NTCP from the germline. In this model, all hepatocytes would be vulnerable to HBV infection and tolerance to the humanized macaque NTCP would be naturally generated. Although much more difficult, costly, and time-consuming to create, this transgenic rhesus macaque HBV model has the potential to answer important questions pertaining to HBV pathogenesis and completely revolutionize testing of HBV therapeutics.

## 5. Conclusions

Given the global health burden of HBV infection, innovative therapies and a better understanding of HBV persistence is imperative. Although many aspects of the virus can be studied using readily available in vitro cell culture models, complex questions need to be addressed within the context of a new immunocompetent HBV in vivo animal model. Here, we outline the current effort to find such an animal model using the newest data on NTCP as the host factor for HBV infection in distinct species and the preliminary success to establish macaques as a promising HBV animal model.

## Figures and Tables

**Table 1:** Factors involved in the early steps of the hepatitis B virus (HBV) replication cycle and their inhibitors.

Step	Protein	Function	Inhibitor
Liver transport	Apolipoprotein E (ApoE)	Liver directed transport along the lipoprotein remnant pathway [13]	None available
Hepatocyte attachment	Heparan sulfate proteoglycans (glypican 5)	Low-affinity attachment of the antigenic loop of both the S protein domain a well as the PreS1 of the L protein [7,14,15]	Synthetic anti-lipopolysaccharide peptides (SALPs) [16]
Receptor binding	Sodium taurocholate co-transporting polypeptide (NTCP)	Hepatocyte-specific HBV take-over through high-affinity binding of the PreS1 domain [17]	Myrcludex B [18], ezetimibe [19], cyclosporin A [20] and derivates [21,22], ibersartan [23], ritonavir [24], rosiglitazone [25], zafirlukast [25], triax [25], sulfasalazine [25], troglitazone [26] and vanitaracin A [27]
Entry	Epidermal growthfactor receptor (EGFR)	Binding of the HBV-NTCP complex and initiation of endocytosis [28]	Gefitinib [28] and erlotinib [29]
Endocytosis	Clathrin and dynamin	Clathrin-mediated endocytosis of the HBV-NTCP-EGFR complex [30,28]	Pitstop-2 [30] and dynasore [31]

**Table 2:** Regions of NTCP crucial for HBV binding and infection.

Species	Common Name	AA 84–87				AA 157–165									Source
Homo sapiens	Human	R	L	K	N	K	G	I	V	I	S	L	V	L	NP_003040
Pan paniscus	Chimpanzee	R	L	K	N	K	G	I	V	I	S	L	V	L	XP_003824149
Tupaia chinesis	Treeshrew	P	L	N	N	V	G	I	V	I	S	L	I	L	XP_006171565
Mus musculus	Mouse	H	L	T	S	K	G	I	M	L	S	L	V	M	NP_001171032
Sus scrofa	Pig	R	L	N	N	G	S	I	V	I	S	L	I	L	XP_001927730
Saimiri boliviensis	Squirrel monkey	Q	L	N	K	G	G	I	M	I	S	L	I	L	XP_003924529
Sapajus apella	Capuchin monkey	R	L	N	K	G	G	I	M	I	S	L	I	L	XP_032101672
Macaca fascicularis	Cynomolgus macaque	Q	L	N	N	G	R	I	I	L	S	L	V	P	NP_001270252

Gray boxes indicate differences from the human (reference) sequence.
